# E-Cigarette Exposure Alters Neuroinflammation Gene and Protein Expression in a Murine Model: Insights from Perinatally Exposed Offspring and Post-Birth Mothers

**DOI:** 10.3390/genes15030322

**Published:** 2024-03-01

**Authors:** Christina Awada, Antonio F. Saporito, Judith T. Zelikoff, Catherine B. Klein

**Affiliations:** Division of Environmental Medicine, New York University Grossman School of Medicine, New York, NY 10010, USA; antonio.saporito@nyulangone.org (A.F.S.); judith.zelikoff@nyulangone.org (J.T.Z.); catherine.klein@nyulangone.org (C.B.K.)

**Keywords:** electronic cigarettes, neuroinflammation, vaping

## Abstract

The use of E-cigarettes, often considered a safer alternative to traditional smoking, has been associated with high rates of cellular toxicity, genetic alterations, and inflammation. Neuroinflammatory impacts of cigarette smoking during pregnancy have been associated with increased risks of adverse childhood health outcomes; however, it is still relatively unknown if the same propensity is conferred on offspring by maternal vaping during gestation. Results from our previous mouse inhalation studies suggest such a connection. In this earlier study, pregnant C57BL/6 mice were exposed daily to inhaled E-cig aerosols (i.e., propylene glycol and vegetable glycerin, [PG/VG]), with or without nicotine (16 mg/mL) by whole-body inhalation throughout gestation (3 h/d; 5 d/week; total ~3-week) and continuing postnatally from post-natal day (PND) 4–21. As neuroinflammation is involved in the dysregulation of glucose homeostasis and weight gain, this study aimed to explore genes associated with these pathways in 1-mo.-old offspring (equivalent in humans to 12–18 years of age). Results in the offspring demonstrated a significant increase in glucose metabolism protein levels in both treatment groups compared to filtered air controls. Gene expression analysis in the hypothalamus of 1 mo. old offspring exposed perinatally to E-cig aerosols, with and without nicotine, revealed significantly increased gene expression changes in multiple genes associated with neuroinflammation. In a second proof-of-principal parallel study employing the same experimental design, we shifted our focus to the hippocampus of the postpartum mothers. We targeted the mRNA levels of several neurotrophic factors (NTFs) indicative of neuroinflammation. While there were suggestive changes in mRNA expression in this study, levels failed to reach statistical significance. These studies highlight the need for ongoing research on E-cig-induced alterations in neuroinflammatory pathways.

## 1. Introduction

Since the advent of modern battery-powered nicotine delivery systems in 2003 [1], electronic cigarettes (E-cigs) and their use patterns have been rapidly evolving [2]. By 2007, when E-cigs were commercially introduced to the United States market, their use for vaping had reached epidemic proportions among young adults and adolescents.

Moreover, many pregnant adult smokers have turned to E-cig use because of their misplaced beliefs regarding the safety of E-cigs (compared to traditional cigarettes) during this vulnerable time. Although the health consequences of exposure to nicotine and smoke products through combustible cigarettes are well documented, less is known about the health effects of generated aerosols via electronic delivery systems (ENDS). Yet, limited research exists concerning the neurotoxic effects of E-cigs, especially among pregnant women and their offspring, who are vulnerable individuals due to their nutritional needs, immune immaturity, and ongoing organogenesis. Previous epidemiologic research [3,4] has shown that E-cig use is adversely linked to increased risks of cardiovascular, pulmonary, neurological, and neurodevelopmental health effects. In addition, in vitro and in vivo laboratory research is actively investigating individual E-cig constituents and e-liquid mixtures and the effects of the use of varied types of vaping devices to derive mechanistic explanations for the observed adverse health outcomes [5,6,7].

Studies performed in this laboratory [8,9] have previously demonstrated the neurotoxicity and brain-inflammatory properties of exposure to E-cig aerosols (with and without nicotine) on the juvenile offspring of exposed pregnant mice. Archived frozen brain sections from exposed post-birth mothers and offspring collected in the Lauterstein et al. (2016) study were used for the investigations described herein [9].

In this study, we first investigated the potential risk of maternal vaping on the increased risk of obesity and dysregulation of metabolic outcomes in the offspring. Epidemiological studies have confirmed that cigarette smoking during pregnancy is linked to overweight or obese offspring from adolescence into adulthood [10,11]. Neural pathways, specifically those in the hypothalamus, are involved in nutritional, hormonal, and neural signals that regulate body weight and glucose homeostasis [12]. During the fetal development of hypothalamic circuits that control metabolism, it is believed that they can be influenced by environmental exposures, including E-cigs. In an in vivo study that investigated the effects of long-term maternal cigarette smoke exposure during pregnancy on hypothalamic appetite regulators, the results revealed obesity, glucose intolerance, and insulin resistance among the female adult offspring [13]. It remains unknown whether E-cig use leads to similar outcomes. In this study, we examined a panel of genes associated with glucose homeostasis in the hypothalamus of offspring exposed perinatally to E-cig aerosols. The genes studied included: Peroxisome proliferator-activated receptor gamma (*PPAR*γ), Leptin receptor long isoform (*LpRb*), Melanocortin-4 Receptor (*MC4R*), AMP-activated protein kinase (*AMPK*), Proopiomelanocortin (*POMC*), and Solute carrier family 2 member 1 (*SLC2A1*). The importance of this study is related to the recent rise in obesity in the United States to levels indicating an epidemic; the prevalence of obesity in the United States is reported at 12.7% among 2-to-5-year-olds, 20.7% among 6–11-year-olds, and 22.2% among 12–19-year-olds [14].

In our second study reported herein, we explored the impact of E-cig exposure during pregnancy on neuroinflammatory effects in the brains of the E-cig-exposed mothers. Neurotrophic factors (NTFs) are a family of proteins that induce the survival, development, and function of neurons, including brain-derived neurotrophic factor (BDNF), neurotrophin-3 (Ntf3), and nerve growth factor (NGF) [15]. These peptides have significant survival-promoting effects, and their dysregulation has been long implicated in neurodegenerative diseases such as Alzheimer’s disease [16] and mental health disorders such as bipolar disorder [17]. However, NTFs also play an increasingly emerging role in chronic disease pathways such as obesity, such that NGF and BDNF have been described among a family of metatrophic factors involved in glucose homeostasis [18]. Cytokines are key regulatory peptide mediators that play critical roles in communications between the immune and central nervous systems. Cytokines primarily involved in neuroinflammation include interleukins (IL-6, Interleukin-1β, [IL-1β]), tumor necrosis factor-α (TNF-α), and C-reactive protein (CRP) [19]. In this portion of the study, expression of NTF and cytokine genes including *TNF-α*, *IL-6*, *IL-1B*, *CRP*, *Ntf3*, *NGF*, and *BDNF* were examined in the post-birth mothers’ hippocampus samples. Inflammation in the hippocampus, mediated by NTF neuropeptides, is critical to the development of obesity-associated neuropsychiatric diseases such as eating disorders, anxiety, and depression. These mood and cognitive alterations are implicated in a bidirectional link between obesity and neuropsychiatric status and can promote and/or predict the development of later-life obesity [20,21]. Thus, this part of the study that examined hippocampal NTFs and cytokines in the E-cig-exposed mothers is relevant to further understanding the role of environmental exposure-induced brain inflammation, which can potentially impact developing neural pathways involved in regulating homeostasis and cell signaling correlated with metabolic disorders [22].

Environmental toxicants, such as particulate matter (PM) and heavy metals (e.g., lead), both found in cigarettes, can exacerbate inflammatory responses that are integral in neurodegenerative or chronic disease etiology [23]. Given many of the similarities between traditional cigarettes and E-cigs (e.g., the presence of nicotine and a variety of other toxic chemicals), it is important to understand whether E-cig aerosol exposures, particularly during gestation, impact neuroinflammatory pathways. Understanding the effects of E-cig exposure on fetal development and offspring health is of great concern, as nicotine (and many of the other aerosol products, including aldehydes and fine-size particulate matter [PM_2.5_]), can cross the placenta and concentrate in fetal blood, amniotic fluid, and/or breast milk [24]. Since E-cig use and obesity have both reached epidemic levels, the primary objective of this dual-part study was to examine the effects of E-cig exposures in neuroinflammatory and neurometabolic pathways in both perinatally exposed offspring and in their directly exposed mothers to establish proof-of-principle concepts for informing future research. This dual-part study, not meant to be comparative in nature, explores the effects of inhaled E-cig aerosol exposure on two distinct populations to determine the potential for E-cig aerosol to alter gene and protein expression associated with neuroinflammation and obesity.

## 2. Materials and Methods

### 2.1. Animal Mating and Exposures

Frozen tissues from C57BL/6 mice—collected during brain dissections from our previous E-cig inhalation exposure study [9]—were used for these investigations. In the original inhalation exposure experiment, male and female C57BL/6 mice (dana), an inbred mouse strain commonly used for neurometabolic studies, were housed individually in polycarbonate cages in a pathogen-free facility (NYU Sterling Forest, NY, USA) and maintained on a 12 h light-dark cycle at 25 °C with a 50% relative humidity. All mice had *ad libitum* access to standard Purina 5001 chow (Purina, St. Louis, MO, USA) and acidified tap water. Mice were acclimated for at least 1-week before exposure. All animal procedures were conducted under NYU Institutional Animal Care and Use Committee (IACUC) approval.

For mating, mice were paired over 4 days, and females were examined daily for the presence of a vaginal plug. After pairing, the males were removed, and females (2/cage) were exposed to either E-cig aerosols (with or without nicotine) or filtered air. The pregnant dams were then isolated and exposed via whole-body inhalation to E-cigs aerosols, with or without nicotine, starting at the appearance of the vaginal plug (gestational day 1, GD 1); dams were separated into single cages and housed individually at or about gestational day GD5, and daily exposures continued until between GD 19–21 (time of birth). After birth, nursing dams and their pups were again exposed to the same E-cig aerosol treatment as during gestation, beginning at PND4–6 and continuing throughout lactation (a total of ~3 weeks).

### 2.2. E-Cig Exposures

**Figure 1:**
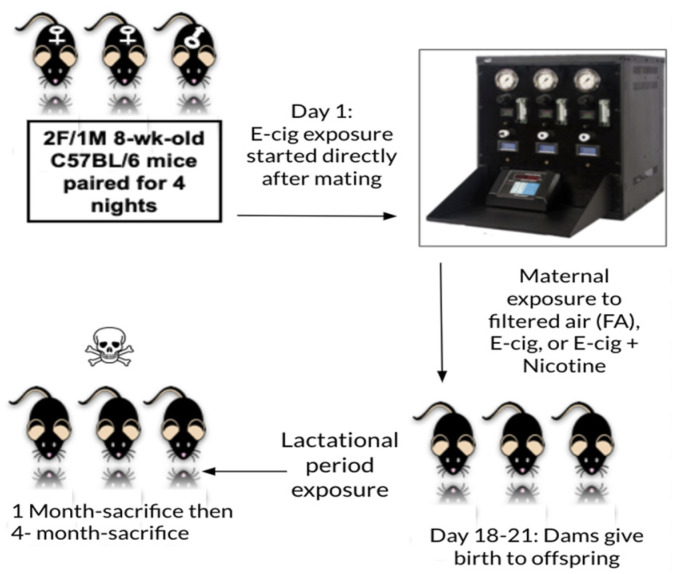
Overall experimental design. FA-filtered air; E-cig, and E-cigarette aerosols with and without nicotine.

As shown in Figure 1, an automated 3-port E-cig aerosol generator (Eaerosols, LLC, Central Valley, NY, USA) was used to produce aerosols from Blu E-cig cartridges (blucigs.com, Edinburgh, Midlothian, UK). As reported previously [9], puff aerosols were introduced into the exposure chambers using charcoal-/HEPA-filtered carrier air that was passed through a rotor-less and brushless diaphragm pump. The puff profile was 35 mL of puff volumes of 4-s duration at 30-s intervals. Each puff was mixed with filtered dilution air before entering the chamber.

For each exposure of pregnant dams, a new classic tobacco flavor cartridge with nicotine (13–16 mg/mL) or a classic tobacco flavor cartridge containing no nicotine was used. The generator voltage was adjusted to maintain a consistent amperage of about ~0.93 Amp delivered to each cartridge. The electrical resistance of each cartridge was measured before insertion to ensure the integrity of the heating coil [9]. During pregnancy, the dams (2/cage) were exposed (whole body) to E-cig aerosols (with or without nicotine) for 3 h/d; 5 days/week in separate 1 m^3^ flow-through exposure chambers. After parturition (beginning at PND 4–6), nursing dams and their pups were co-exposed together in the same cage. During each short exposure, food and water were removed from the cages to avoid contamination; both food and water were available immediately after exposure.

### 2.3. Tissue Processing

Approximately 4–6 days after the final exposure, offspring (now at PND 25–31) and dams in all treatment groups were euthanized by intraperitoneal injection of 120 mg pentobarbital/kg (Sleepaway; Fort Dodge Labs, Fort Dodge, IA, USA). At necropsy, whole brains were removed from each male and female offspring and post-birth dams and dissected to separately section and archive the frontal cortex, hippocampus, hypothalamus, midbrain, striatum, cerebellum, and olfactory bulb sections. Each brain tissue sample was snap-frozen in liquid N_2_ and stored at −80 °C until used for future experiments [9]. For the current studies, the hypothalamus of each offspring and the hippocampus sections of the post-birth dams were retrieved from liquid N_2_, thawed, and used for gene expression and protein analyses. In the case of the offspring, hypothalamic samples from the offspring were pooled in mixed-sex batches (per treatment group) to acquire sufficient quantities of protein and mRNA [25] for the evaluation of each treatment group.

### 2.4. Western Blotting

For Western blotting, hypothalamus tissues from the offspring were pooled in mixed-sex groups of 3 offspring per treatment group and processed using Abcam kits (Cambridge, UK). Pooling of hypothalamic tissues of both sexes was necessary due to the tiny size of the 4-week-old offspring brain sections; thus, comparisons between sexes could not be carried out for these studies. Tissue samples were transferred into radioimmunoprecipitation assay buffer (RIPA; 150 mM NaCl, 1% TritonX-100, 0.5% sodium deoxy-cholate, 0.1% SDS, 50 mM Tris [pH 8.0]) and homogenized with a glass pestle. The recovered materials were transferred to sterile 15-mL centrifuge tubes, boiled, and then centrifuged at 12,000× *g* (10 min at room temperature). The resulting supernatants were collected, and protein concentrations were quantified using a Pierce BCA Protein Assay Kit (Thermo Scientific, Waltham, MA, USA). An aliquot of total protein (30 μg) from each sample was resolved over a 12% gel using sodium dodecyl sulfate-polyacrylamide gel electrophoresis (SDS-Page) and subsequently electrotransferred to a nitrocellulose membrane (Cell Signaling, Boston, MA, USA). For each protein examined, a separate membrane was generated to avoid subsequent issues of membrane stripping and handling impacts on antigen integrity (Abcam, Cambridge, UK).

Each membrane was blocked for 1 h (at 32 °C) with 5% non-fat milk in Tris-buffered saline (pH 7.6) containing 0.1% Tween-20 (TBST). The membranes were then incubated overnight (at 4 °C) with an appropriate primary antibody (at a 1:1000 dilution, as per the manufacturer’s recommendation) in 5% non-fat milk in TBST buffer. The primary rabbit antibodies used for this part of the study included: glucose transporter-1 (GLUT1), α-amino-3-hydroxy-5-methyl-4-isoxazole propionic acid receptors (AMPAR), peroxisome proliferator-activated receptor γ (PPARγ), and glyceraldehyde 3-phosphate dehydrogenase (GAPDH; loading control; Cell Signaling, Boston, MA, USA). Each membrane was washed three times in TBST, then coated with a solution of 5% non-fat milk-TBST buffer containing horseradish peroxidase (HRP)-conjugated anti-rabbit IgG (1:100 dilution, as per manufacturer’s recommendation [Cell Signaling, Boston, MA, USA]). Membranes were then incubated for 1 h at room temperature and washed three times with TBST. Expression of target protein bands was visualized using Signal-Fire ECL Reagent (Cell Signaling, Boston, MA, USA), as per the manufacturer’s instructions.

### 2.5. RNA Isolation

Hypothalamic RNA from 4 offspring of mixed sexes (per treatment group) was pooled and processed using the TRIZOL protocol (Invitrogen, Carlsbad, CA, USA) of Chomczynski (1995) [26]. Again, the tiny size of the hypothalamus sections (thus providing extremely limited tissue) did not allow for individual sex-matched analysis. For the post-birth dams, RNA was extracted from archived hippocampus tissue samples (per exposure [n = 2–4 samples/batch]). Isolated RNA samples were dissolved in DEPC-treated water, and RNA concentration and purity were quantified using a NanoDrop 2000/2000c System (Thermo Fisher, Waltham, MA, USA). One μL from each exposure sample was treated with 5 μL of 2X reverse transcription buffer and 1 μL of 20X RT Enzyme Mix (Applied Biosystems, Carlsbad, CA, USA). Samples were then briefly vortexed to spin down the contents and eliminate any air bubbles before initiating the reverse transcription reactions.

### 2.6. Reverse Transcription-Quantitative Polymerase Chain Reaction (RT-qPCR) Analysis

qPCR analyses were performed (QuantStudio Pro 6 System, Thermo Fisher Scientific) in triplicate on the hypothalamus tissue of the offspring and hippocampus tissue of the dams using PowerUp™ SYBR™ Green Master Mix (Thermo Fisher Scientific, Waltham, MA, USA). Primers were designed from previous studies [27,28,29,30,31,32,33] or using the NCBI Primer-BLAST tool [23,27] and obtained from Thermo Fisher Scientific (Table 1 and Table 2). The genes examined for offspring hypothalamus expression include Peroxisome proliferator-activated receptor gamma (*PPAR*γ), Leptin receptor long isoform (*LpRb*), Melanocortin-4 Receptor (*MC4R*), AMP-activated protein kinase (*AMPK*), Proopiomelanocortin (*POMC*), and Solute carrier family 2 member 1 (*SLC2A1*). Genes studied in the post-birth dam hippocampus samples included *TNF-α*, *IL-6*, *IL-1B*, *CRP*, *Ntf3*, *NGF*, and *BDNF*. Each specific gene was chosen due to its significance in the specific brain region examined, either for the hippocampus or hypothalamus, as well as for their potential role in networks associated with metabolic disorders, including neuroinflammatory-mediated obesity.

In all cases, the primers and RT-qPCR conditions used for amplifications were pre-optimized using linear efficiency tests for each gene of interest (Thermo Fisher Scientific, Waltham, MA, USA). The thermocycler protocol was set for 95 °C melt for 3-min, followed by 40 cycles of 95 °C melt for 10-s, 57–60 °C annealing for 30-s, and a 10-s extension step at 72 °C. mRNA levels for each gene of interest were normalized for Glyceraldehyde 3-phosphate dehydrogenase (*GAPDH*) gene expression using a 2^−ΔΔCt^ method [34].

### 2.7. Statistical Analysis

Protein signal bands were quantified using Image J 1.52a software (NIH, Bethesda, MD, USA). All protein data were normalized to GAPDH levels on each gel to permit statistical comparisons between the treatment groups. Statistical analyses were performed using GraphPad Prism 8 (GraphPad Software Inc., San Diego, CA, USA). Western blot data are reported with means ± SEM values, which were calculated from three independent experiments. The normality of the data were determined by Shapiro–Wilk testing. To assess statistical significance, unpaired two-tailed Student’s *t*-tests or one-way analysis of variance (ANOVA) were employed for group comparisons, with *p*-values denoted by * *p* < 0.05.

RT-qPCR fold change data are shown in terms of mean ± SEM. The normality of the data were determined by Shapiro–Wilk tests. Parametric fold change data generated from RT-qPCR analyses between the treatment groups were determined using ANOVA, followed by Bonferroni post-hoc testing, if appropriate. Non-parametric fold change data were analyzed using Kruskal-Wallis tests. Differences were considered significant when *p*-values were <0.05. All statistical analyses were performed using Prism software (v.8.0, GraphPad, San Diego, CA, USA).

## 3. Results

### 3.1. Offspring: Western Blot Analyses of Select Proteins in Offspring Hypothalamus

**Figure 2:**
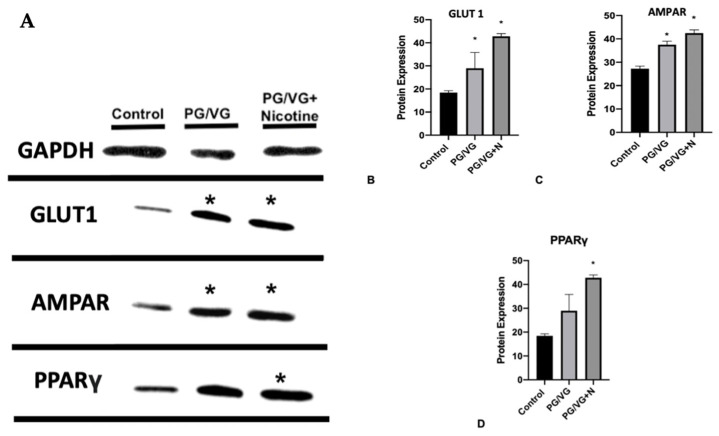
(**A**) Western blot expression bands from pooled hypothalamic samples of male and female offspring exposed perinatally to E-cig aerosols (with and without nicotine) were densitometrically quantified, and comparisons to filtered air offspring levels were then performed. The data shown are means ± SEM. * *p* < 0.05 vs. control. In all cases, n = 3 samples/treatment group. (**B**) Hypothalamic GLUT-1 expression, (**C**) Hypothalamic AMPAR expression, (**D**) Hypothalamic PPARγ expression, and Western blot expression bands from pooled hypothalamic samples of male and female offspring exposed perinatally to E-cig aerosols (with and without nicotine) were densitometrically quantified, and comparisons to filtered air offspring levels were then performed. The data shown are means ± SEM. * *p* < 0.05 vs. control. In all cases, n = 3 per treatment group.

Perinatal exposure to E-cig aerosols, with or without nicotine, altered hypothalamic protein levels in ~1-month-old offspring (mixed sex, three/treatment group), compared to those of the filtered air controls. A representative Western blot (Figure 2) of 1-month-old offspring exposed to E-cig aerosols, with and without nicotine, revealed significantly increased protein levels (*p* < 0.05) of GLUT-1 and AMPAR compared to levels measured in age-matched filtered air control offspring (Figure 2A,B). Glut1 and AMPAR are implicated in the complex interplay between neuroinflammation and obesity. Glut1, by mediating glucose transport, contributes to metabolic processes that can influence obesity-related pathways. In the context of neuroinflammation, increased Glut1 expression has been associated with inflammation in the central nervous system. On the other hand, AMPAR, being involved in excitatory neurotransmission, can modulate neural circuits that regulate appetite and energy balance. Dysregulation of AMPAR function has been linked to neuroinflammatory processes as well as alterations in synaptic plasticity, which may contribute to both obesity and neuroinflammation. Concerning PPARγ expression, juvenile offspring exposed to E-cig aerosols (PG/VG) with nicotine demonstrated a significant increase in protein levels (*p* < 0.05) (Figure 2C), compared to E-cig aerosols (PG/VG) without nicotine and the filtered air control.

### 3.2. Perinatally Exposed Offspring: RT-qPCR Analysis of Selected Genes in Hypothalamus Tissues following Exposure to E-Cig Aerosols

**Figure 3:**
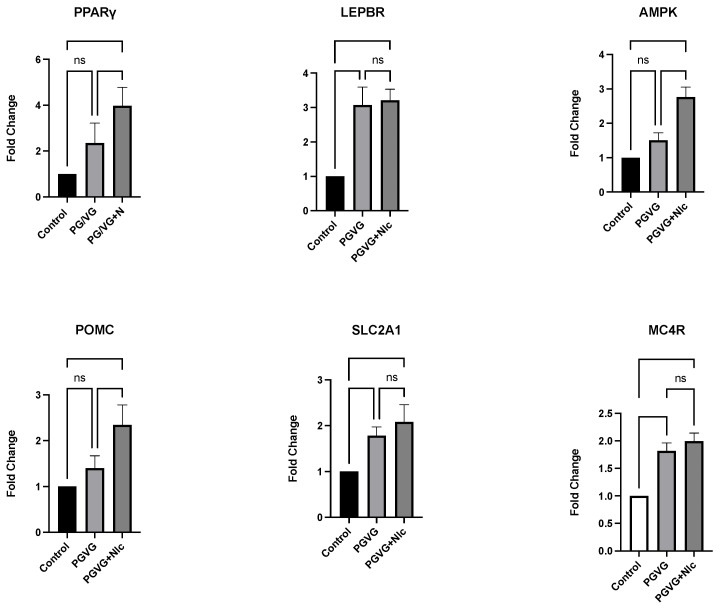
Mean ± SEM gene fold changes calculated by one-way ANOVA for *PPARγ*, *LepRb*, *MC4R*, *AMPK*, *POMC*, and *SLC2A1*, in the hypothalamus samples of offspring exposed perinatally to E-cig aerosols (with and without nicotine), compared to filtered air controls (n = 4 per treatment group).

Real-time RT-qPCR analyses of mRNA from the hypothalamus tissues revealed that exposure to E-cig aerosols, with and without nicotine, altered the expression of certain genes in ~1-mo-old, perinatally exposed offspring, compared to the age-matched, air-exposed controls. Analysis of six genes involved in neural pathways, i.e., *PPARγ*, *LepRb*, *MC4R*, *AMPK*, *POMC*, and *SLC2A1*, revealed significant (*p* < 0.05) alterations in the expression levels of each gene in offspring exposed to E-cig aerosols, with and without nicotine (Figure 3 and Table 3).

Specifically, compared to air control values, there was a 2.3-fold increase in *PPARγ* in offspring exposed to E-cig aerosols (PG/VG) without nicotine and a 3.9-fold increase (from control) in offspring exposed to E-cig aerosols with nicotine. A 3.2- and 3.1-fold increase in expression of *LepRb* (compared to air controls) was observed in offspring perinatally exposed to E-cig aerosols, both with and without nicotine, respectively. Expression of *MC4R* was increased by 1.9-fold and 1.8-fold (compared to controls) in offspring exposed to E-cig aerosols, with and without nicotine, respectively. *AMPK* expression was increased 2.7-fold in offspring exposed to E-cig aerosols with nicotine and increased 1.5-fold in the E-cig treatment group without nicotine. Similarly, offspring exposed perinatally to PG/VG + nicotine showed a 2.3-fold increase in *POMC* and a 1.4-fold increase without nicotine, compared to air controls. Finally, 2.1-fold and 1.8-fold increases (compared to control) in *SLC2A1* expression were observed in response to E-cig exposures, with and without nicotine, respectively.

### 3.3. Post-Birth Dams: RT-qPCR Analysis of Selected Genes in Hippocampal Tissues following Exposure to E-Cig Aerosols

**Figure 4:**
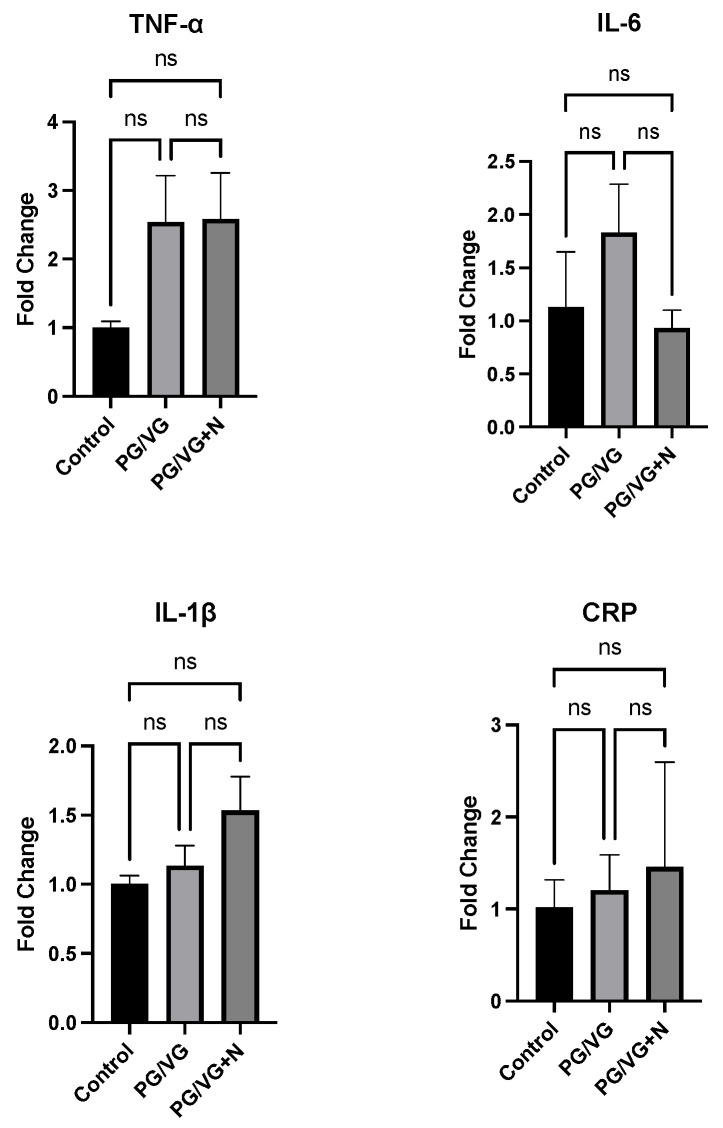
Mean ± SEM gene fold changes calculated by one-way ANOVA for pro-inflammatory cytokines *TNF-α*, *IL-6*, *IL-1β*, and the upstream regulating protein *CRP*, in the hippocampi samples of dams exposed to E-cig aerosols (with and without nicotine), compared to filtered air controls (n = 4 and n = 5, respectively/treatment group).

**Figure 5:**
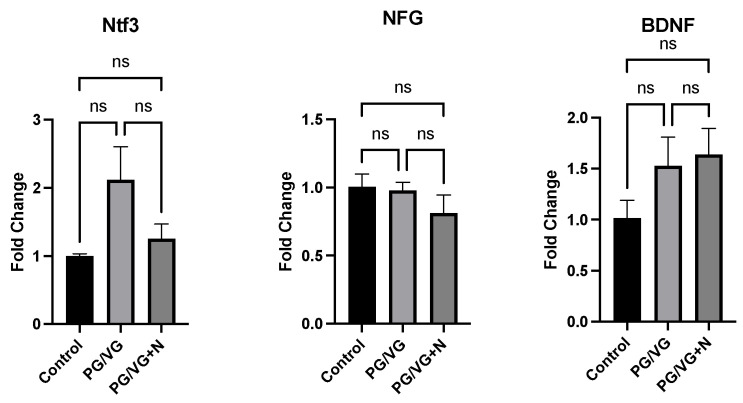
Mean ± SEM gene fold changes calculated by one-way ANOVA for the neurotrophic factors, *Ntf3*, *NGF*, and *BDNF* in the hippocampi samples of dams exposed to E-cig aerosols (with and without nicotine), compared to filtered air controls (n = 4 and n = 5, respectively/treatment group).

RT-qPCR analyses of hippocampal tissues of the dams revealed modest differences (although not statistically significant) in gene expression for *TNF-α*, *IL-6*, *IL-1b*, *CRP*, *NtF3*, *NGF*, and *BDNF* (Figure 4) between the treatment groups (with or without nicotine) compared to their age-matched, air-exposed counterparts. Specifically, fold-increases (compared to the control group) for *TNF-α* expression were 2.54 and 2.58 for the PG/VG and PG/VG + nicotine groups, respectively. *TNF-α* expression analysis showed especially large standard deviation values (>1.0), indicating high variability in fold changes for both aerosol treatment groups. For *IL-6*, the mean fold changes compared to control values (1.13) were increased 1.83-fold for the PG/VG group and decreased 0.93-fold for the PG/VG + nicotine group. C-reactive protein (*CRP*) expression levels were increased 1.20-fold and 1.45-fold (compared to controls) for the PG/VG and PG/VG + nicotine treatment groups, respectively. Interleukin 1-β (*IL-β*) had similar fold-changes with increases of 1.13 and 1.53, respectively, compared to controls. For the NTF genes of interest, no significant (<0.05) differences were observed between any treatment group (Figure 5). Mean fold changes for *NGF* (compared to controls) were calculated at 0.98 and 0.81 for the PG/VG and PG/VG + nicotine E-cig exposure groups, respectively. Although none of the differences between e-cig treatment groups reach statistical significance in post-birth dams, using one-way ANOVA, *BDNF* gene expression fold changes increased to 1.53 and 1.63 for PG/VG and PG/VG + nicotine, respectively, compared to the 1.02 average fold change calculated for the age-matched air control group. Lastly, the expression of *Ntf3* increased in the PG/VG exposure group (fold change of 2.12), compared to the control group (1.0). However, due to a large standard deviation in the PG/VG exposure group, differences between the groups failed to reach statistical significance. Thus, exposure of directly exposed dams to E-cig aerosols (with or without nicotine) failed to produce any significant effects (*p* < 0.05) on the examined pro-inflammatory cytokines, or NTF genes of interest.

## 4. Discussion

This study hypothesized that perinatally exposed offspring and their mothers directly exposed during pregnancy and lactation to E-cig aerosols, with or without nicotine, lead to protein and/or gene expression changes associated with neuroinflammatory pathways in the hypothalamus of juvenile offspring and the hippocampus of post-birth dams. Maternal exposure to some toxicants during sensitive windows of fetal development can alter developmental processes that are essential for fetal growth and development [35]. Such exposures could permanently alter metabolic growth, development, and/or cognition in the offspring during early life that could be carried into adulthood, potentially leaving those individuals predisposed to later-life chronic diseases. Our proof-of-principle findings herein with the perinatally exposed offspring underscore the notion that early-life E-cig exposures have the potential to alter neuroinflammatory genes, reprogram metabolism, and potentially lead to downstream risks of such adversities as obesity, diabetes, and neurological disorders in the offspring as they age.

### 4.1. Metabolic Dysregulation, Obesity, and Diabetes in Offspring

Inflammation’s pivotal role in obesity has been emphasized by Motawi et al. (2017) [36]. The investigators suggested that variations in lung inflammation between obese and lean individuals could be due to the influence of PPARγ. Furthermore, it has been reported that PPARγ expression in the hypothalamus acts as a metabolic sensor, controlling cellular processes like fatty acid and glucose metabolism [36]. In this study, gene expression of PPARγ in hypothalamus samples from juvenile offspring exposed perinatally to E-cig aerosols with nicotine was significantly upregulated compared to that observed in filtered air control samples.

GLUT1 protein levels and gene expression in the offspring hypothalamus tissues were also increased in pups exposed perinatally to E-cig aerosols, with or without nicotine, in this study. This, in turn, highlights a likely increased affinity for glucose and possible glucose uptake, which can play a role in the progression of diseases like diabetes. Nutritional excess has the potential to induce acute inflammation at the molecular level within the hypothalamus. For instance, in vivo studies have demonstrated that a sudden oversupply of glucose or lipids can trigger inflammation in the hypothalamus, manifesting within a span of a few hours to three days [37]. These observations imply that hypothalamic inflammation may precede and, consequently, serve as a contributing factor to diseases induced by overnutrition. Energy utilization in the brain requires communication between different GLUT family proteins, as they are critical components of cellular “crosstalk” mechanisms [38]. Furthermore, increased gene expression of the solute carrier family 2-member 1 (*SLC2A1*) gene, which encodes glucose transporter-1, was also observed in the juvenile (1-mo.) offspring perinatally exposed to E-cig aerosols, with or without nicotine. These results support the findings observed in this study by Western blot analysis and discussed above.

Neuroinflammation refers to the inflammation of the brain and nervous tissue, and emerging research suggests its involvement in a variety of neurological disorders, such as Alzheimer’s disease, Parkinson’s disease, depression, and eating disorders [39]. Thus, it is crucial to consider the potential implications of neuroinflammation in the context of perinatal exposure to E-cig aerosols. Studies have indicated that exposure to environmental factors, such as E-cig aerosols, may trigger inflammatory responses within the central nervous system [40,41]. Such responses involve the activation of immune cells in the brain, the release of inflammatory mediators, and subsequent changes in the neural microenvironment. In the context of the hypothalamus, where an increase in AMPAR protein levels was observed in offspring in the present study, neuroinflammation could further exacerbate excitatory signaling and contribute to disruptions in normal neural function [42]. Such an alteration could potentially lead to dysregulation in hormonal balance, appetite control, and stress responsiveness, as well as negative alterations in neural circuits governing food intake and body weight [43,44]. Further research in this area will contribute to a better understanding of the complex mechanisms underlying the impact of E-cigs on neural development and function, as well as guide future studies of less well-understood crosstalk between cognitive (hippocampal) brain signals in mothers and developing hypothalamic pathways in their offspring.

The melanocortin system is significant for energy homeostasis and has been shown to regulate food intake, obesity, and weight homeostasis [30]. In the present study, expression of melanocortin system genes *MC4R* and *LepRb* was significantly upregulated in the offspring in both E-cig treatment groups (with or without nicotine) compared to air controls. Mutations inducing loss of function of several genes within this pathway, including mutations in *POMC*, *LepRb*, or *MC4R* genes, have been shown in other in vivo studies to cause early-onset severe obesity [45].

Leptin (via its receptor LepRb) modulates gene expression in hypothalamic-expressing neurons that control energy balance and glucose homeostasis [46]. The increase in *LepRb* expression in the offspring observed in the present study after perinatal exposure to E-cigs (with or without nicotine) could have important implications for normal vs. defective hypothalamic circuits, which, in turn, could adversely impact multiple downstream metabolic mechanisms.

Significant upregulation of *AMPK* and *POMC* gene expression was observed in tissues recovered from offspring perinatally exposed to E-cigs, both with nicotine. The products of these two genes are critical for regulating energy homeostasis at the hypothalamic level [47]. *AMPK* acts as an essential mediator of the central and peripheral effects of many appetite-regulating hormones. In contrast, *POMC* plays a crucial role in energy homeostasis and is secreted from neurons located in the dorsomedial nucleus (DMH) of the hypothalamus [48]. Previous research has linked nicotine exposure with decreased food intake via the expression of receptors for circulating signals, such as leptin and ghrelin, and by activation of *POMC* neurons [49]. As for *AMPK*, nicotine has been shown to increase the phosphorylation of *AMPK* in a time-dependent manner in adipocytes. In an in vitro study by Wu et al. (2015) [50], AMPK activation by nicotine (in differentiated 3T3L1 adipocytes) led to increased lipolysis, suggesting that nicotine-induced activation of *AMPK* can lead to metabolic effects.

Taken together, the results of the present study could suggest a disturbance in the central energy homeostasis in the hypothalamus of the offspring. It could also be suggested that E-cig exposure is not harmless and that the impacts on neuroinflammation need to be more fully investigated.

### 4.2. Neuroinflammation in Exposed Offspring

In juvenile offspring exposed perinatally to E-cig aerosols with nicotine, results revealed a significant increase in hypothalamic expression of the nuclear hormone receptor, *PPARγ*. The activation of PPARγ assumes a pivotal role in governing various aspects of the central nervous system (CNS), including proliferation, metabolism, differentiation, development, and inflammatory responses [51]. Within the brain, *PPARγ* activation has demonstrated its ability to impede the synthesis and release of pro-inflammatory cytokines, and its inhibition in the CNS is characterized by pronounced inflammation, potentially leading to long-term chronic disease [52]. Pro-inflammatory cytokines can also regulate synaptic strength, with AMPAR playing an essential role in synaptic plasticity [53]. Specifically, TNF-α can induce significant alterations in neurotransmission by modifying AMPAR subunits [54]. In brain slices from neonatal mice, Bliss et al. (2011) demonstrated that TNF-α induced dose-dependent neuronal excitotoxicity was achieved through increased activity of calpains in Purkinje neurons [55]. Additionally, TNF-α has the capacity to inhibit the activity of glutamate transporters, consequently augmenting neurotoxicity, which is relevant to the significantly increased AMPAR protein levels observed in the perinatally exposed offspring in this study in response to E-cig exposure, with and without nicotine.

### 4.3. Systemic Inflammation: Inflammation in Exposed Post-Birth Dams

Another critical factor in disease development is the role of systemic inflammation. Inflammatory cascades can act as mediators and catalysts in the progression of chronic diseases [56]. Production of inflammatory proteins such as PPARγ, TNF-α, IL-6, IL-10, and CRP in the central nervous system is associated with sleep regulation, aging, and disease progression [57]. Inflammatory-related diseases include arthritis [58], Alzheimer’s disease [59], and diabetes [60], where the overproduction of inflammatory proteins contributes to disease etiology. Previous mouse studies have even shown that hippocampal dysregulation of pro-inflammatory cytokines can further disease progression in patients with obesity [61]. Unexpectedly, in this study, no statistically significant differences (compared to control mice) were observed in the expression of pro-inflammatory cytokines in the hippocampi of post-birth mothers exposed both during gestation and lactation (30 days after birth).

While fold changes in genes of interest such as *TNF-*α exhibited increased expression in the hippocampus of both E-cig exposure groups, neither value in the post-birth dams reached statistical significance compared to air-exposed controls. Other genes in the same host tissue, including *IL-6*, while also not statistically significant (compared to controls and PG/VG alone), could suggest a reduction in gene expression following exposure to PG/VG + nicotine. However, it is important to note that the portion of the study investigating post-natal dams served primarily as a proof-of-principle study using only a small group of dams. Therefore, future studies need to rely on a larger cohort of mice to elucidate the potential health implications of E-cig aerosol exposure on post-partum dams.

The original study by Lauterstein et al. (2016) from which this study stemmed, examining frontal cortex and hippocampus gene expression changes in male and female juvenile offspring exposed perinatally to E-cig aerosols, investigated the role of a variety of inflammatory pathway proteins [9]. These included neuroreceptors such as nerve growth factor receptor (*Ngfr*), glial cell line-derived neurotrophic factor (*Gdnf*), α-1D adrenoreceptor (*Adra1d*), and other proteins such as galanin (gal) and choline O-acetyltransferase (*Chat*). In contrast to the significant differences in gene expression observed in offspring hippocampi in that study (i.e., *Chat* and *Adra1d* were significantly increased in 4-months-old male offspring following perinatal E-cig exposure, with and without nicotine), such findings were not observed in the archived hippocampus tissues recovered from the post-birth dams. Based on our knowledge of vulnerable stages of life, especially during critical windows of development, future studies should focus on the sensitivity of different gestational stages to investigate the effects of E-cig on specific neurodevelopmental biomarkers and pathways, including potential cross-talk between neurocognitive and hypothalamic processes. This could lead to more targeted interventions and regulations to protect the health of both developing progeny and adult populations.

### 4.4. Cognitive and Metabolic Dysfunction in Post-Birth Dams

In this study, neuroinflammatory pathways associated with metabolic dysfunction that could influence cognitive function were also examined in post-birth mothers. NTFs play a critical role in the survival of sympathetic and sensory neurons, as well as their maintenance, differentiation, and growth [62]. Differences in their expression could lead to the development of psychiatric diseases or pathological aging [63]. Dysregulation of BDNF, in particular can lead to impaired activation of tyrosine kinase receptor β (TrkB), resulting in obesity, insatiable appetite, and reduced energy expenditure [64]. Furthermore, the bidirectional relationship between NTFs and chronic disease may offer a therapeutic route for metabolic dysregulation, as increased BDNF levels may reduce obesity and diabetes [65]. Nevertheless, in this small proof-of-principle part of the study, NTF genes (i.e., *BDNF*, *NGF*, or *Ntf3*) from the hippocampi of post-birth dams exposed directly by inhalation to E-cig aerosols during pregnancy and lactation exhibited no significant differences amongst or between any E-cig treatment groups (Figure 5). However, slight increases in the expression of *Ntf3*, a critical human gene associated with diabetic neuropathy [66], were observed in dams exposed to PG/VG compared to control mice. The significance of such a trend requires further study. Given the biological importance of NTF genes, such as *BDNF*, which appears to have a relationship with psychiatric diseases [67,68], investigation of the impacts of E-cig exposure on NTF expression with a larger cohort is important to further clarify potential effects.

## 5. Conclusions

This study investigated alterations in neuroinflammatory pathways in the hypothalami of perinatally exposed progeny resulting from exposure to E-cig aerosols, with and without nicotine. The current findings suggest that E-cig aerosol exposure, using vaping conditions reflective of human scenarios, throughout gestation and most of lactation leads to alterations in some metabolic functions and other metabolic parameters of neuroinflammatory pathways in the offspring that can be associated with the potential development of obesity. Specific proteins associated with metabolic dysregulation were also adversely impacted in offspring perinatally exposed to E-cig aerosols. These same offspring also exhibited increased expression of six genes whose alterations in expression are known to be associated with changes in metabolic outcomes that could be associated with disease. In the second part of this study, neuroinflammation and cytokines were examined in the hippocampi of the E-cig-exposed mothers. The results showed no significant differences in inflammatory marker expression of several NTFs or of pro-inflammatory cytokines. With the rising popularity of E-cigs as an effective nicotine delivery system, an urgent call for more research into their short- and long-term effects is warranted, particularly for pregnant women and their unborn and newborn children. Taken together, the results from this study reveal that early life exposure to E-cig aerosols, with or without nicotine, could lead to alterations in the offspring’s hypothalamic proteome and genome, which could affect a variety of metabolic pathways and cognitive functions associated with neuroinflammation. The E-cig-induced effects in the offspring that occurred in the absence of nicotine point to the importance of other toxic aerosol constituents in bringing about some of the observed adverse outcomes. Overall, these two proof-of-principle studies indicate that E-cig exposure early in life adversely affects the neuroinflammation process and pathways essential for neurological health in the offspring. Moreover, they also suggest that nicotine is not necessarily the driving E-cig component responsible for alterations in brain health and that components other than nicotine pose a potential health risk, particularly during pregnancy.

## 6. Limitations and Future Directions

As with all studies, the present study is not without limitations. The hypothalamic samples from the offspring were pooled into mixed-sex batches, thus not allowing for observation of sex-dependent differences in response to E-cig exposures. However, the Authors feel that each portion of the study stands on its own to inform the health implications of these two separate populations. We recognize that, from a comparative viewpoint, future studies should concentrate on one area of the brain in various gestational windows and life stages to reach more definitive comparative conclusions. Further studies should also consider the role of E-cig exposure on epigenetic regulation of gene expression, which can be critical for fetal programming and placental development and impact later life health and/or disease.

## Figures and Tables

**Table 1:** RT-qPCR genes of interest (5′ to 3′) for the hypothalamus of offspring exposed to E-cig aerosol.

Gene of Interest	Forward Sequence	Reverse Sequence	References
PPARγ	CGGTTTCAGAAGTGCCTTG	GGTTCAGCTGGTCGATATCAC	[28]
LepRb	AATGCGAATGTCATGTAGCAG	ACTCCAGTCACTCCAGACTCC	[29]
MC4R	GGAAGATGAACTCCACCCACC	AATGGGTCGGAAACCATCGTC	[30]
AMPK	CATGGCTGAGAAGCAGAAGCAC	CTTAACTGCCACTTTATGGCCG	[31]
POMC	TTCCAGTATGACTCCACTCACG	AGACTCCACGACATACTCAGCA	[32]
SLC2A1	CCAGCTGGGAATCGTCGTT	CAAGTCTGCATTGCCCATGAT	[33]

**Table 2:** RT-qPCR Genes of Interest (5′ to 3′) for the hippocampus of dams exposed to E-cig aerosol.

Gene of Interest	Forward Sequence	Reverse Sequence	Reference
GAPDH	GTGGCAAAGTGGAGATTGTTG	CGTTGAATTTGCCGTGAGTG	[27]
TNF-α	CTACCTTGTTGCCTCCTCTTT	GAGCAGAGGTTCAGTGATGTAG	
IL-6	GTCTGTAGCTCATTCTGCTCTG	GAAGGCAACTGGATGGAAGT	
IL-1B	GGTGTGTGACGTTCCCATTA	ATTGAGGTGGAGAGCTTTCAG	
CRP	GCCTTTCACTTCTCTGCTTTG	GAGTCCTAGTGGGATGCTTATG	
Ntf3	CCTGGAAATAGTCACACGGATG	CTTGGATGCCACGGAGATAAG	
NGF	CAGTGAGGTGCATAGCGTAAT	CTCCTTCTGGGACATTGCTATC	
BDNF	CTGAGCGTGTGTGACAGTATTA	CTTTGGATACCGGGACTTTCTC	

**Table 3:** Summary of gene expression changes observed in the hypothalamus of offspring exposed to E-cig aerosols. (N/A = not applicable).

Genes of Interest in the Hypothalamus of Offspring	Gene Expression (Upregulated or Downregulated)	
	PG/VG	PG/VG + Nic
PPARγ	N/A	Upregulated
LepRb	Upregulated	Upregulated
MC4R	Upregulated	Upregulated
AMPK	N/A	Upregulated
POMC	N/A	Upregulated
SLC2A1	Upregulated	Upregulated

## Data Availability

Data is contained within the article.
